# Case Report: Cardiac Surgery and Combined Lipid-Lowering Drug Therapy for Homozygous Familial Hypercholesterolemia

**DOI:** 10.3389/fped.2020.535949

**Published:** 2020-10-22

**Authors:** Mingxin Gao, Wenyuan Yu, Hui Hu, Hongli Liu, Kangjun Fan, Chengxiong Gu, Lvya Wang, Yang Yu

**Affiliations:** ^1^Department of Cardiac Surgery, Beijing Anzhen Hospital, Capital Medical University, Beijing, China; ^2^Department of Cardiology, Beijing Anzhen Hospital, Capital Medical University, Beijing, China

**Keywords:** cardiac surgery, valvular heart disease, coronary heart disease, dyslipidemia, homozygous familial hypercholesterolemia (HoFH)

## Abstract

Homozygous familial hypercholesterolemia (HoFH) is a rare, autosomal dominant, hereditary, metabolic disease. HoFH patients exhibit severe coronary stenosis and valvular disease, which may result in sudden death, even during adolescence. The challenges faced during surgery and the poor curative effect of conventional lipid-lowering therapy create a treatment bottleneck. We report a rare case of HoFH in a 12-years-old boy with acute myocardial infarction, severe mitral insufficiency, and moderate aortic insufficiency. Coronary artery bypass grafting and valvuloplasty resulted in improved heart function. Postoperative combined lipid-lowering drug therapy was able to reduce low-density lipoprotein cholesterol level from 15.37 mm/L to 6.41 mmol/L. Thus, the combination of medical and surgical treatment was considered effective and can be used to inform treatment guidelines for HoFH with severe complications.

## Introduction

Homozygous familial hypercholesterolemia (HoFH) is a rare, autosomal dominant, hereditary, metabolic disease with a prevalence rate of 1 in 160,000–300,000 people worldwide (1). Abnormally elevated low-density lipoprotein cholesterol (LDL-C) levels, which are usually >13 mmol/L, can lead to cutaneous xanthoma, coronary heart disease, valvular disease, aortic dysplasia, and multiple arterial stenoses throughout the body. HoFH patients are relatively young, have severe coronary stenosis, and are at risk of sudden death (2). Surgical treatment for HoFH is challenging and conventional lipid-lowering therapy has poor curative effect, owing to a defect in the LDL-C receptor in these patients. We report a case of HoFH in a child with severe coronary stenosis and valvular disease who underwent successful treatment via surgery and the administration of combined lipid-lowering drug therapy.

## Case Description

A 12-years-old boy presented with no history of hypertension or diabetes. Initial symptoms included angina pectoris following activity beginning 7 months before presentation whereas myocardial ischemia and valvular insufficiency were subsequently diagnosed at a local hospital 4 months before presentation. FH was suspected based on a history of xanthoma identified in his 35-years-old mother, while his 36-years-old father showed no symptoms. His laboratory results at presentation were as follows: total cholesterol (TCHO), 16.14 mmol/L; triglyceride (TG), 1.57 mmol/L; and LDL-C, 15.37 mmol/L. Rosuvastatin (Shandong Pharmaceutical Co. Ltd., China, 20 mg/tablet) was administered as the preliminary lipid-lowering therapy; however, the myocardial ischemia and valvular insufficiency progressed from mild to severe within 4 months, and his cardiac function, according to the New York Heart Association Classification, was identified as grade IV. Electrocardiography (ECG) was performed, indicating acute non-ST-segment elevation myocardial infarction. A coronary angiogram revealed 85, 95, and 99% stenosis of the left main artery, ramus ostia, and right coronary initial segment, respectively; the Syntax Score was 62 points. The entire coronary artery lumen diameter was <1 mm, the left ventricular end-diastolic diameter was 48 mm, and the left atrium diameter was 44 mm. The ejection fraction was 68%. Two large eccentric regurgitation signals from the A1 and A3 areas were observed in the left atrium (reflux area: 7.7 cm^2^). The central regurgitation area in the aortic valve was 5.2 cm^2^ and the aortic annulus diameter was 15.5 mm. The following laboratory results were determined at baseline: TCHO, 15.04 mmol/L; TG, 1.44 mmol/L; and LDL-C, 13.21 mmol/L. Aortic computed tomography-angiography indicated that the aortic arch and wall vessels were thickened, with an increase in the surrounding density. A color Doppler ultrasound of the carotid vertebral and subclavian arteries indicated 47% stenosis of the right common carotid artery, 47% stenosis of the right internal carotid artery, and diffuse thickening of the right subclavian artery.

## Diagnostic Assessment

Rosuvastatin and ezetimibe (Hubei Moke Chemical Co., Ltd., China, 10 mg/tablet) were administered as the preliminary lipid-lowering therapy at a dosage of 20 and 10 mg/day, respectively, but had a limited effect after 1 week. At this point, the laboratory test results were as follows: TCHO, 13.78 mmol/L; TG, 3.51 mmol/L; and LDL-C, 11.45 mmol/L. Thus, probucol (Qilu Pharmaceutical Co. Ltd, China, 0.125 g/tablet), at a dosage of 0.5 g twice daily, was added to the drug regimen in week 2. Next-generation sequencing was conducted and identified two low-density lipoprotein receptor (LDLR) point mutations in the patient: Cys329Tyr (c.986G>A), and Trp490Arg (c.1468T>C). Reverse-cascade genetic screening was performed on the patient's parents and the LDLR point mutations were confirmed to be inherited from his mother and father (Cys329Tyr, and Trp490Arg, respectively). These two point mutations are known and are listed on the online Leiden Open Variation Database (https://databases.lovd.nl/shared/variants/LDLR). HoFH was diagnosed based on the next-generation sequencing results; consequently, evolocumab, a proprotein convertase subtilisin/kexin type 9 (PCSK9) inhibitor (Amgen, USA, 140 mg/ml) was added to the regimen at week 3 at a dosage of 420 mg monthly. The effects of the latter drug regimen could not be evaluated because of the scheduled surgery.

**Figure 1:**
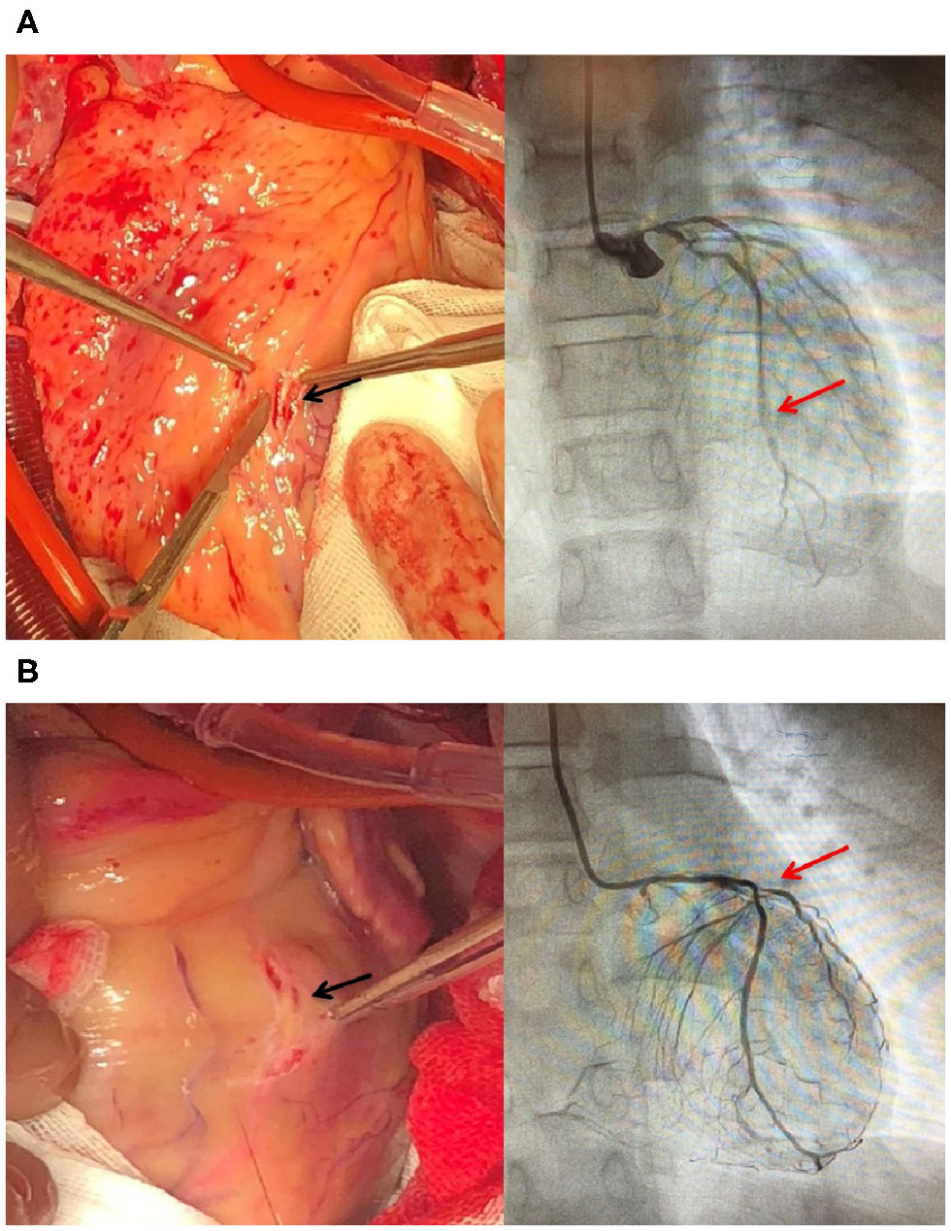
Severely stenosed coronary arteries in an HoFH patient. **(A)** Calcified plaques (black arrow) in the middle of the left descending artery in the heart of the patient. Stenosis (red arrow) in the middle of the left descending artery. **(B)** Calcified plaques (black arrow) in the initial ramus. Stenosis (red arrow) in the initial ramus.

**Figure 2:**
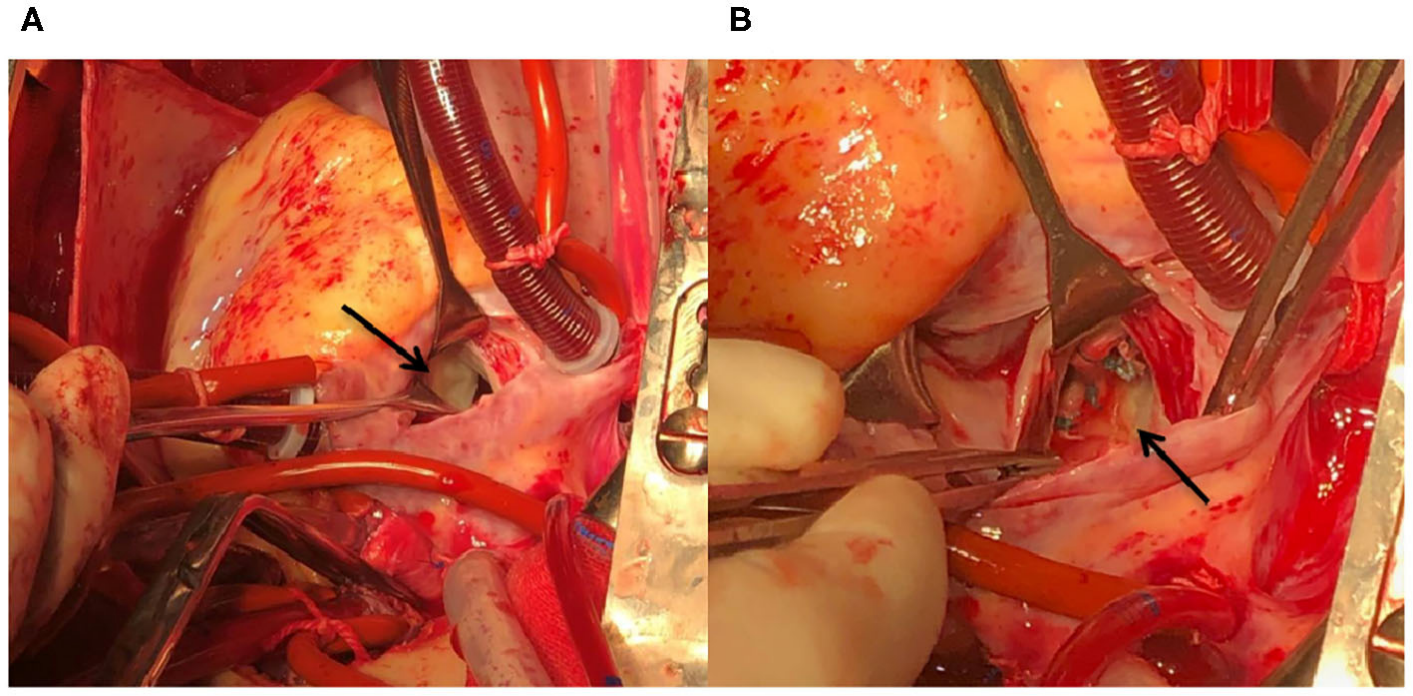
HoFH affecting the structure and function of the mitral valve device. **(A)** Poorly matched A3 and P3 areas (black arrow). **(B)** An ED4600 mitral annuloplasty ring is sutured to the mitral ring after edge-to-edge repair of the A1–P1 and A3–P3 areas (black arrow).

**Figure 3:**
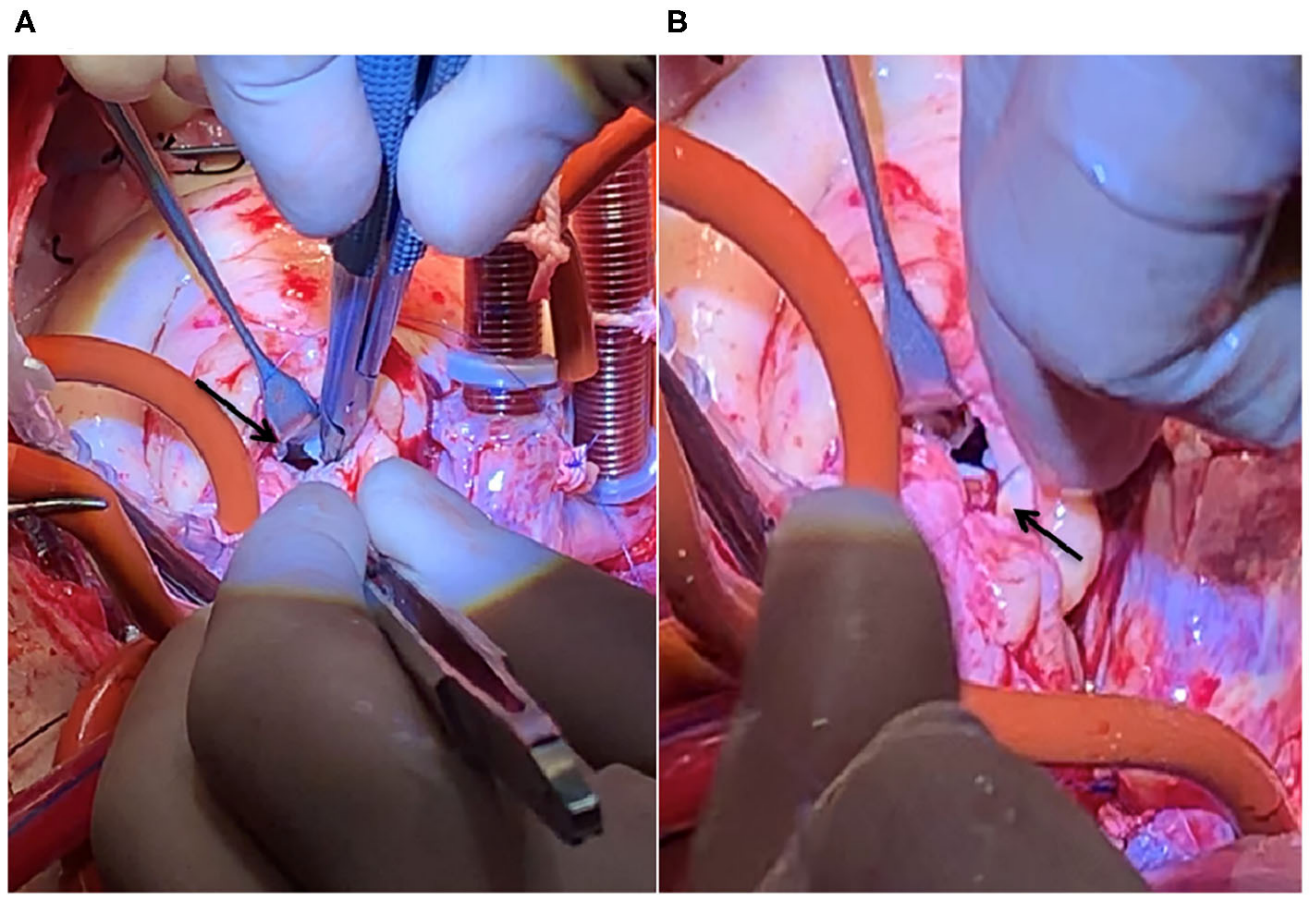
HoFH affecting the structure and function of the aortic valve device. **(A)** The left coronary valve is slightly drooped (black arrow). **(B)** 5-0 Prolene line for clamping the autologous pericardium and suturing the junction of the left and non-crown flaps (black arrow).

Coronary artery bypass grafting (CABG) was performed via the great saphenous vein to the left anterior descending branch and the great saphenous vein to the ramus to the posterior left ventricular branch using an 8-0 Prolene line (Johnson & Johnson, USA), under a beating heart. Severe calcification was also observed in the left anterior descending branch and lumen of the ramus (Figure 1). The mitral valves in the A1–P1 and A3–P3 areas under the cardiopulmonary bypass were poorly joined; thus, after edge-to-edge repair, ED4600 (AG Edwards & Sons, Inc., USA) mitral annular contraction was used to connect the two regions (Figure 2). The modified DeVega procedure was performed on the tricuspid valve, and an autologous pericardial patch was mattress-sutured to the slightly drooped left coronary valve using a 5-0 Prolene line (Figure 3). Micro-reflux was observed in both valves during a water injection test.

No complications occurred postoperatively, no abnormalities were identified on ECG, and only mild aortic and mitral regurgitation was observed. On postoperative day 1, laboratory test results were as follows: TCHO, 7.81 mmol/L; TG, 1.79 mmol/L; and LDL-C, 6.69 mmol/L. Rosuvastatin (20 mg/day), ezetimibe (10 mg/day), probucol (0.5 g twice daily), and a PCSK9 inhibitor (420 mg/month) were administered as combined lipid-lowering drug therapy.

**Figure 4:**
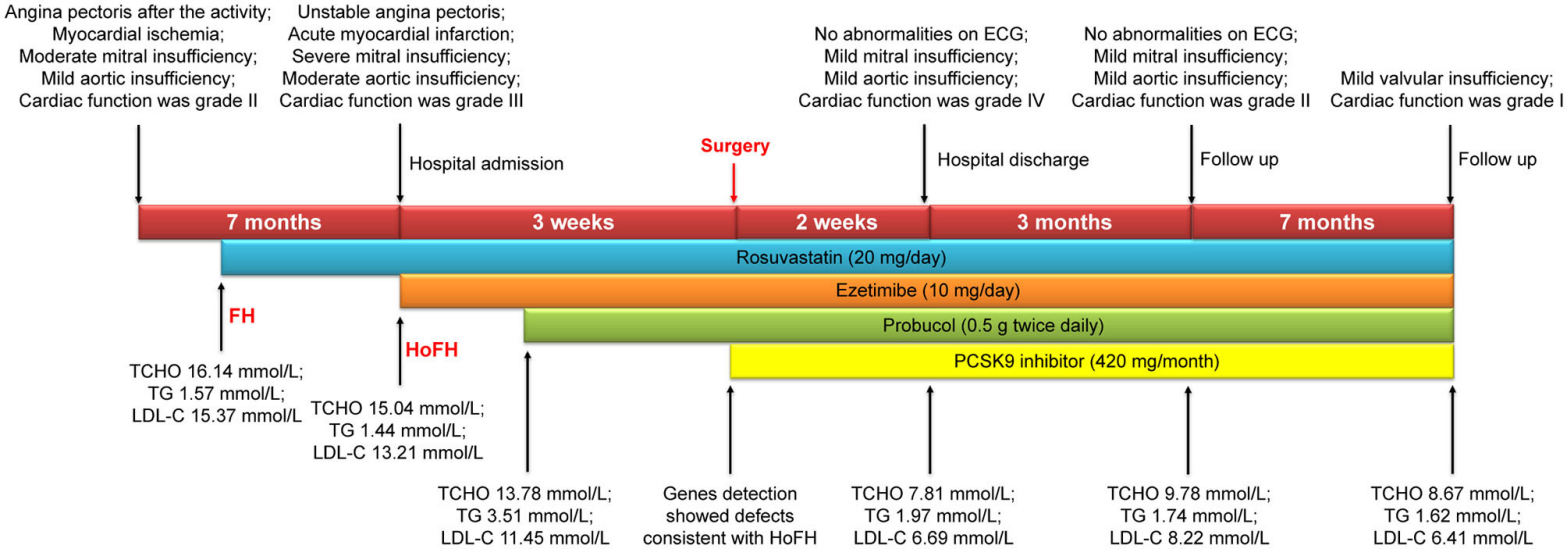
Timeline of the patient's entire diagnosis and treatment.

At the 3-months follow-up, his ECG appeared to be normal and an echocardiogram revealed an ejection fraction of 76%, a left ventricular end-diastolic diameter of 28 mm, a left atrium diameter of 34 mm, and mild aortic and mitral regurgitation. His laboratory results were as follows: TCHO, 9.73 mmol/L; TG, 1.74 was mmol/L; and LDL-C, 8.22 mmol/L. His cardiac function grade improved to grade II. The 7-months follow-up echocardiogram indicated mild valvular insufficiency; TCHO was 8.67 mmol/L, TG was 1.62 mmol/L, and LDL-C was 6.41 mmol/L. Cardiac function grade had further improved to grade I. A timeline detailing the course of the patient's diagnosis and treatment is shown in Figure 4.

All procedures performed were in accordance with the ethical standards of the institutional research committee and the 1964 Helsinki Declaration and its later amendments, and the patient's parents provided consent for publication of his data.

## Discussion

HoFH patients with severely high LDL-C levels are relatively young and have cardiovascular abnormalities (3). We showed that surgery can effectively cure coronary artery and valve disease, thereafter, providing the opportunity to control the primary disease effectively using combined lipid-lowering drug therapy.

Because HoFH patients have high LDL-C levels at birth, usually >13 mmol/L, untreated patients often develop coronary artery disease before the age of 20 and die before they turn 30 (4). Since 2001, our hospital has managed 80 HoFH families; among the affected family members, the average age of the patients was 15.0 years while the average untreated LDL-C level was 13.246 mmol/L. Among these families, 11% had myocardial infarctions, 45% had coronary artery plaques in the ostia, 37% had aortic insufficiency, and 52% had mitral insufficiency (5). The parents of the patient in the present case were also found to have severe coronary stenosis, massive mitral regurgitation, and moderate aortic regurgitation. Therefore, performing surgery was necessary for the survival of our patient.

There are few reports on the management of HoFH patients and the use of surgery to treat HoFH cases in the literature. We encountered two issues during surgery. The first was dysgenesis of the coronary artery; the diffuse stenosis was expected to increase the perioperative myocardial infarction risk (6). Second, there was no suitable mechanical or biological valve for the aortic valve annulus, owing to its small diameter of 15.5 mm. Thus, valvuloplasty was performed instead of valve replacement and the child was discharged without complications thereafter.

A reduction in the LDL-C level by 1 mmol/L reduces the vascular event risk by 21% and all-cause mortality by 10% in the mildly hypercholesterolemic population (7). For children with HoFH, the LDL-C level should decrease to <3.4 mmol/L, or by >50% of the original level (8); however, this cannot be achieved via the use of statins alone (10–25%), or in combination with ezetimibe (20–40%) (9). A PCSK9 inhibitor, a new lipid-lowering drug, directly prevents the interaction of PCSK9 with the LDL receptor, reduces the decomposition of the LDL receptor, mediated by PCSK9, and enhances the recycling of the receptor and LDL-C scavenging, which further reduces LDL-C levels by 31% compared with the reduction that can be achieved by the use of statins alone (10). Lomitapide is an inhibitor of the microsomal triglyceride transfer protein, which can reduce LDL-C levels by around 40% in HoFH patients undergoing treatment with statins, either with or without LDL apheresis. However, side effects including gastrointestinal symptoms and the production of liver fat, and lomitapide is not licensed by the China Food and Drug Administration (11). However, because most drugs increase the LDLR level by upregulating LDLR expression, structural defects of the LDLR limit the efficacy of those drugs because they do not alter receptor function (12). In our experience, the maximum tolerated dose of statins is the first choice of therapy; ezetimibe combined with statins makes up the combined lipid-lowering therapy if the LDL-C level is above the standard; the PCSK9 inhibitor is an intensive lipid-lowering treatment that can be used based on gene sequencing results; plasmapheresis can be performed if necessary; and probucol is a potent hypolipidemic drug that regresses xanthoma formation and carotid atherosclerosis, in conjunction with a marked reduction in HDL-C levels (13). In the present case, the LDL-C level was high (13.21 mmol/L) after rosuvastatin administration alone. When rosuvastatin was administered in combination with ezetimibe, the LDL-C level slightly decreased to 11.45 mmol/L. Probucol was subsequently added to the regimen and the PCSK9 inhibitor was added before surgery. After performing the cardiopulmonary bypass, LDL-C ultimately decreased by 50% from the baseline. The A rebound in LDL-C levels from 6.69 to 8.22 mmol/L was observed at the 3-months follow-up because the cardiopulmonary bypass, similar to plasmapheresis, could only lead to a transient reduction in LDL-C levels. However, at the 7-months follow-up, the LDL-C levels had once again lowered to 6.41 mmol/L. These findings highlight the necessity of combined lipid-lowering drug therapy in HoFH patients.

Currently, the HoFH diagnostic rate is <1%. Mortality and disability rates are high because of cardiovascular events (14); thus, most HoFH patients do not have the opportunity to undergo lipid-lowering drug therapy after cardiovascular events occur. Active intervention by surgeons is therefore required to improve outcomes and facilitate further postoperative testing of combined lipid-lowering drug therapies.

There are some limitations to this case report. First, we can only report on the 7-months follow-up results of this patient; however, we are continuing his follow-up. Second, we have not explored the effects of surgery and combined lipid-lowering drug therapy in more patients. Thus, further research with a larger sample size is required to confirm the effects of this treatment regimen in HoFH patients. Third, although LDL-C levels were reduced by >50% of the original level, they remained outside the normal range for healthy individuals. Therefore, evinacumab, an inhibitor of angiopoietin-like protein 3, could be added to the treatment regimen to reduce this patient's LDL-C independent of the defect in the LDL receptor (15). Liver transplantation may be the radical cure of HoFH for this patient as a last option.

In conclusion, we successfully performed a CABG and valvuloplasty in a child with HoFH, with no complications. Combined lipid-lowering drug therapy was provided both during the perioperative period and postoperatively. The 7-months follow-up results were remarkable, with improvements in both cardiac function and serum lipid levels. We therefore believe that surgery will allow physicians to effectively control the primary disease via combined lipid-lowering drug therapy in HoFH patients.

## Patient Perspective

The patient and his parents received substantial information regarding the disease and the treatment modalities from the doctors; the parents consented to all surgical and drug therapies.

## Data Availability Statement

All datasets generated for this study are included in the article/supplementary material.

## Ethics Statement

The studies involving human participants were reviewed and approved by this case was approved by the Institutional Review Board of Beijing An Zhen Hospital of Capital Medical University. Written informed consent was obtained from the minors' legal guardians (parents) for the publication of any potentially identifiable images or data included in this article.

## Author Contributions

MG, LW, and YY conceived and designed the study. MG, WY, HL, and KF were responsible for the administration of the perioperative treatment and examinations. LW conducted the gene testing and was responsible for the combined lipid-lowering drug therapy. MG, HH, CG, and YY performed the surgery. All authors read and approved the manuscript.

## Conflict of Interest

The authors declare that the research was conducted in the absence of any commercial or financial relationships that could be construed as a potential conflict of interest.

## Abbreviations

CABG: coronary artery bypass grafting
ECG: electrocardiography
HoFH: homozygous familial hyperlipidemia
LDL-C: low-density lipoprotein cholesterol
LDLR: low-density lipoprotein receptor
PCSK9: proprotein convertase subtilisin/kexin type 9
TCHO: total cholesterol
TG: triglyceride.
